# Functional and Bioactive Performance of Premixed Bioceramic Sealers with Warm Obturation: A Scoping Review

**DOI:** 10.3390/gels11110932

**Published:** 2025-11-20

**Authors:** Patryk Wiśniewski, Stanisław Krokosz, Małgorzata Pietruska, Anna Zalewska

**Affiliations:** 1 Department of Periodontal and Oral Mucosa Diseases, Medical University of Bialystok, 15-269 Bialystok, Poland; 2 Department of Restorative Dentistry, Medical University of Bialystok, 15-089 Bialystok, Poland; 3 Independent Laboratory of Experimental Dentistry, Medical University of Bialystok, 15-089 Bialystok, Poland

**Keywords:** premixed bioceramic sealers, warm gutta-percha, calcium silicate, interface bioactivity, hydroxyapatite formation, mineralization, functional gels, regenerative endodontics, obturation techniques, periapical repair

## Abstract

Premixed bioceramic sealers represent a recent advancement in endodontic obturation, combining bioactivity, moisture-induced mineralization and favorable handling properties. When used with warm gutta-percha techniques, these calcium silicate-based sealers are exposed to elevated temperatures that may influence their physicochemical behavior and interfacial performance. This review aimed to summarize current evidence on premixed bioceramic sealers used in conjunction with thermoplastic obturation techniques. A comprehensive literature search was conducted in PubMed, Scopus, and Web of Science for studies published between January 2020 and July 2025 evaluating the physicochemical properties, bioactivity, sealing ability, fracture resistance, clinical outcomes and retreatability of premixed bioceramic sealers under warm obturation conditions. No meta-analysis was performed—this review provides a narrative synthesis of the available evidence within this scope. Twenty-five studies met the inclusion criteria. In vitro and ex vivo data indicate that premixed bioceramic sealers generally maintain chemical stability and bioactivity when exposed to clinically relevant heating protocols, with favorable dentinal tubule penetration, interfacial adaptation and the formation of calcium silicate hydrate, and hydroxyapatite at the sealer–dentin interface. These characteristics are associated with improved filling homogeneity, potential reinforcement of root dentin and high rates of periapical healing reported in limited short-term clinical studies. However, the evidence also highlights important challenges, including technique-sensitive retreatability, material remnants after re-instrumentation and concerns regarding overextension, and long-term dimensional stability. Within the limitations of predominantly in vitro and short-term clinical evidence, premixed bioceramic sealers used with warm gutta-percha techniques appear to be promising functional materials that combine mechanical sealing with bioactive and mineralizing potential. Standardized protocols and robust long-term clinical studies are needed to confirm their durability, retreatability and prognostic impact in routine endodontic practice.

## 1. Introduction

Bioceramic sealers have gained significant importance in endodontics due to their high biocompatibility, bioactive properties, and ability to create a tight, long-lasting seal for the root canal system [1]. Initially derived from medical applications, bioceramics were introduced into dentistry in the form of mineral trioxide aggregate (MTA)-based materials and later as components of root canal sealers [2,3]. Traditional bioceramic sealers, which required manual mixing prior to application, exhibited limited thermal resistance and variable handling characteristics, making them unsuitable for use with warm obturation techniques [4].

Recent technological advancements have led to the development of premixed bioceramic sealers, which are supplied as ready-to-use formulations with standardized consistency, enhanced stability, and extended working time. These materials are based on calcium silicate and calcium phosphate compounds dispersed in aqueous vehicles, allowing controlled hydration and ion release during setting. Such formulations exhibit a colloidal, paste-like structure that enables intimate adaptation to dentinal walls and promotes the formation of a hydrated calcium silicate layer and hydroxyapatite at the sealer–dentin interface [5,6]. This bioactive mineral phase supports interfacial bonding and may contribute to the repair of periapical tissues through biologically driven mineralization processes.

The combination of the bioactivity and sealing ability of bioceramic sealers with the three-dimensional filling capacity of warm gutta-percha techniques—such as the continuous wave (CWT) and warm vertical compaction (WVC) methods—has opened new avenues for improving the quality of root canal obturation [7]. This synergistic approach enhances material adaptation, facilitates the filling of lateral canals, and reduces the risk of microleakage, while preserving the regenerative and repairing potential inherent to calcium silicate-based materials.

Despite the growing clinical and scientific interest, the current literature lacks comprehensive evaluations focusing on the practical and biological aspects of this innovative combination of material and technique. Therefore, this systematic review explicitly addresses the following research question: how do premixed bioceramic sealers perform when used in conjunction with contemporary warm gutta-percha obturation techniques? In line with this, the review aims to synthesize available evidence on their physical properties, interfacial behavior and sealing ability under thermoplastic conditions, bioactivity and mineralization at the dentin–sealer interface, and their potential role in functional tissue repair within the broader context of regenerative endodontics.

## 2. Materials and Methods

In order to present a comprehensive overview of premixed bioceramic sealers and warm obturation techniques, a systematic review was undertaken utilizing three electronic databases: Web of Science, Scopus, and PubMed. Google Scholar was excluded due to concerns regarding its limited search capabilities, lack of transparency, and inclusion of non-peer-reviewed literature. The review process adhered to the Preferred Reporting Items for Systematic Reviews and Meta-Analyses (PRISMA) 2020 guidelines [8]. The following search strategies were employed:

1. For Web of Science: TS = (“bioceramic sealer” OR “calcium silicate sealer” OR “calcium silicate-based sealer” OR “BC Sealer” OR “bioceramic material”) AND TS = (“gutta-percha” OR “warm gutta-percha” OR “thermoplastic technique” OR “carrier-based obturation” OR “vertical compaction” OR “Thermafil”) AND TS = (“endodontic treatment” OR “root canal” OR “root canal obturation” OR “endodontics”)
2. For Scopus: (ALL(premixed biosealers) OR ALL(premixed bioceramic sealer) OR ALL(premixed bioceramics) OR ALL(premixed calcium silicate sealer) AND ALL(root canal treatment) OR ALL(RCT) OR ALL(endodontic treatment) AND ALL(warm gutta-percha obturation) OR ALL(warm vertical compaction gutta-percha) OR ALL(thermoplasticized gutta-percha) OR ALL(obtura II) OR ALL(warm condensation)
3. For PubMed: (“bioceramic sealer” OR “calcium silicate sealer” OR “calcium silicate-based sealer” OR “BC Sealer” OR “bioceramic material”) AND (“gutta-percha” OR “warm gutta-percha” OR “thermoplastic technique” OR “carrier-based obturation” OR “vertical compaction” OR “Thermafil”) AND (“endodontic treatment” OR “root canal” OR “root canal obturation” OR “endodontics”)

We included studies researching the use of premixed bioceramic sealers with warm obturation techniques in endodontic root canal treatments published between 2020 and July 2025. The first search was conducted on 2 June 2025; the last search was conducted on 18 July 2025. The search was restricted to 2020–2025 in order to capture the most recent generation of premixed bioceramic sealers specifically developed for use with warm obturation techniques. Earlier studies mainly evaluated older formulations not designed for thermal application and were therefore excluded to ensure clinical relevance and comparability of findings. Exclusions were applied to studies published in different languages than English; pediatric studies; non-diagnostic or prognostic applications; insufficient sample size or poor statistical analysis; non-clinical studies; case reports; reviews; expert opinions and editorials.

Titles and abstracts were independently screened by two reviewers (S.K. and P.W.), followed by full-text assessments to determine eligibility according to predefined inclusion and exclusion criteria. Any disagreements were resolved through consensus with arbitration by a third reviewer (A.Z.) when necessary. Duplicates were identified and manually removed using Zotero reference management software by both reviewers (S.K. and P.W.).

The inclusion and exclusion of studies were guided by the PICO framework, as outlined in Table 1 and Table 2. A detailed description of the search strategy and study selection process is provided in the Section 3, following the PRISMA 2020 protocol [8]. Both in vitro and in vivo studies were included because they provide complementary and clinically relevant information on this material–technique combination: in vitro experiments allow detailed assessment of physicochemical properties, setting behavior, interfacial adaptation, and retreatability under controlled thermoplastic conditions, whereas in vivo studies document periapical healing, postoperative complications, and functional outcomes associated with the clinical use of premixed bioceramic sealers with warm obturation techniques. The review protocol was preregistered on the Open Science Framework (OSF; registration ID: osf.io/n7zef).

### Quality Assessment and Critical Appraisal for the Systematic Review of Included Studies

**Figure 1:**
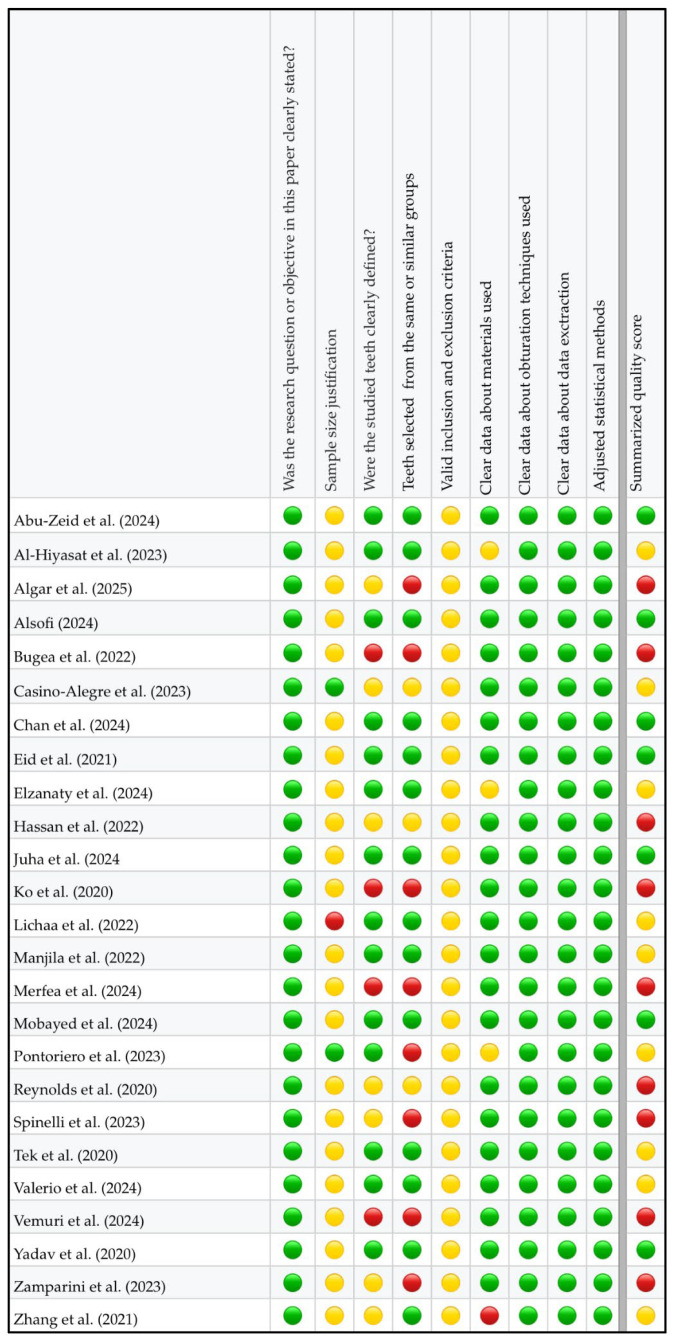
Quality assessment of the included studies based on the National Heart, Lung, and Blood Institute Study Quality Assessment Tools. For each domain, “yes” responses indicating low risk of bias are marked in green, “no” responses or clearly inadequate reporting indicating high risk of bias are marked in red, and unclear or not reported items are marked in yellow. The last column (“summarized quality score”) reflects the overall study quality, categorized as good (green: most key criteria fulfilled, low risk of bias), intermediate (yellow: some concerns or incomplete reporting), or poor (red: multiple domains at high or unclear risk of bias) [10,11,12,13,14,15,16,17,18,19,20,21,22,23,24,25,26,27,28,29,30,31,32,33,34].

‘Study Quality Assessment Tool’ issued by the National Heart, Lung, and Blood Institute, National Institute of Health was used to assess the risk of bias in each of the individual studies included in this review [National Heart, Lung, and Blood Institute. Study Quality Assessment Tools. Available from: https://www.nhlbi.nih.gov/health-topics/study-quality-assessment-tools (accessed on 7 August 2025.)]. Quality assessment questionnaires for each included study were completed independently by two reviewers (S.K. and P.W.). Any disagreements were resolved through discussion, with a third reviewer (A.Z.) serving as arbitrator when consensus could not be reached. A summary of the quality appraisal for each individual study is presented in Figure 1.

The level of evidence for the included studies was evaluated using the Oxford Centre for Evidence-Based Medicine Levels of Evidence. All studies were classified as Level 3 or Level 4 evidence on the five-level scale [9].

## 3. Results

**Figure 2:**
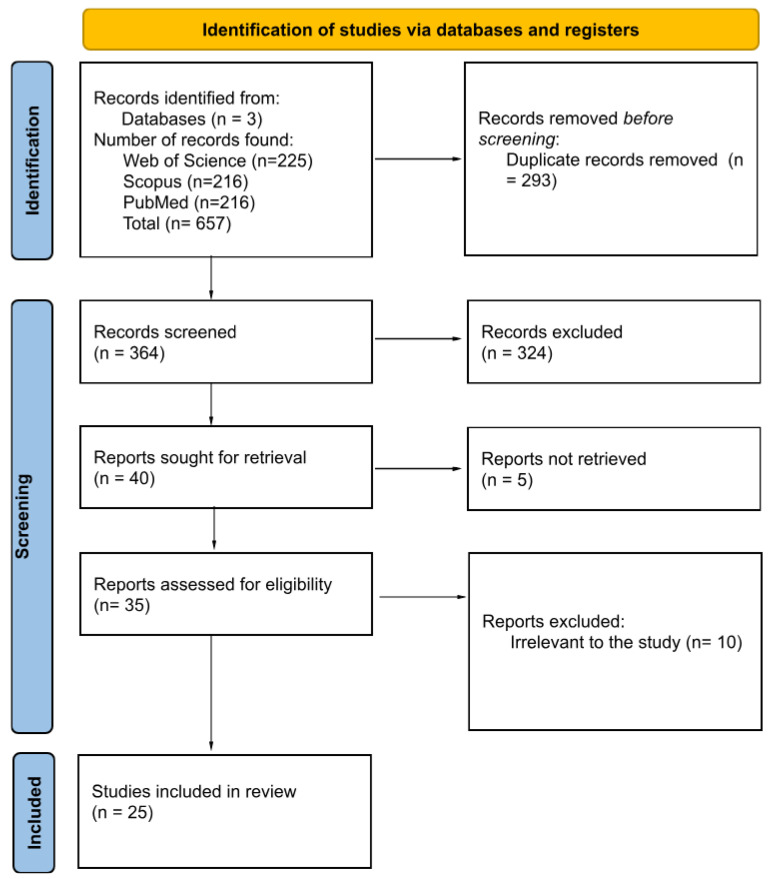
PRISMA 2020 flow diagram [PRISMA. PRISMA 2020 Flow Diagram. Chechlist available in the Supplementary Materials. Available online: https://www.prisma-statement.org/prisma-2020-flow-diagram accessed on 7 August 2025.

**Figure 3:**
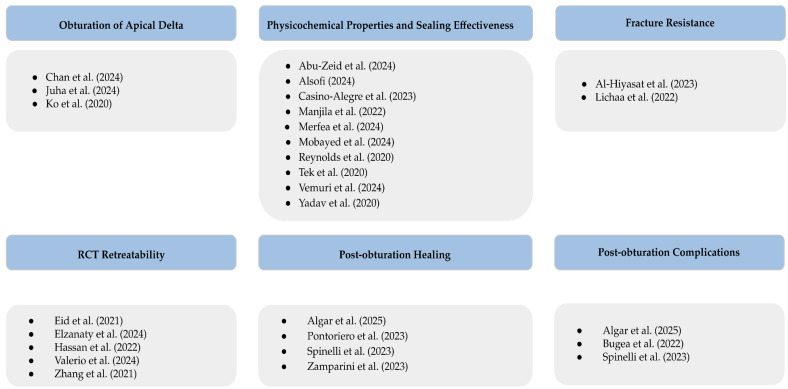
Thematic structure of the literature review presenting the main topics of the analyzed studies (see Table 3, Table 4, Table 5, Table 6, Table 7 and Table 8) [10,11,12,13,14,15,16,17,18,19,20,21,22,23,24,25,26,27,28,29,30,31,32,33,34].

A total of 657 records were initially identified across the PubMed, Scopus, and Web of Science databases. Prior to screening, 293 duplicate entries were removed, resulting in 364 unique publications subjected to title and abstract screening. 324 manuscripts were excluded based on title and abstract relevance. The exclusion of studies was guided by the PICO framework, as outlined in Table 1 and Table 2. Among the remaining 40 records, 5 full-text articles could not be retrieved. Following full-text evaluation of the remaining 35 studies, 25 were deemed eligible and included in this systematic review based on the predefined inclusion and exclusion criteria (see Table 1, Figure 2 and Figure 3).

### 3.1. Physicochemical Properties and Sealing Effectiveness

In the study by Abu-Zeid et al. (2024) [10], the bioceramic sealer EndoSequence BC Sealer HiFlow (Brasseler, Savannah, GA, USA) demonstrated a statistically significantly superior ability to form the thinnest sealer layer and exhibited the highest flowability when compared to a conventional bioceramic sealer EndoSequence BC sealer (Brasseler, Savannah, GA, USA) and a resin-based sealer AH Plus (Dentsply Sirona, Charlotte, NC, USA). These properties were further enhanced when the WVC technique was employed, as opposed to cold obturation methods. In cold obturation techniques, no statistically significant differences were observed in dentinal tubule penetration between the BC and BCH bioceramic sealers; however, the resin-based sealer AH Plus exhibited the lowest degree of dentinal tubule penetration. Conversely, under WVC, the BCH sealer achieved the highest level of dentin penetration, followed by AH Plus, while the conventional BC sealer demonstrated the lowest penetration.

Similar findings were reported in the study by Alsofi (2024) [11], in which the use of the WVC technique in combination with the EndoSequence BC Sealer HiFlow (Brasseler, Savannah, GA, USA) sealer also resulted in the greatest dentinal tubule penetration compared to the AH Plus (Dentsply Sirona, Charlotte, NC, USA) and the conventional EndoSequence BC sealer (Brasseler, Savannah, GA, USA). Moreover, the author confirmed that incubating the BCH sealer in phosphate-buffered saline (PBS) led to the formation of hydroxyapatite crystals, further substantiating the bioactive properties of this material.

The study conducted by Casino-Alegre et al. (2023) [12] aimed to evaluate the ability of the EndoSequence BC Sealer HiFlow (Brasseler, Savannah, GA, USA) sealer to penetrate dentinal tubules within the root canal system. The authors compared the widely used single-cone technique with three warm obturation methods: CWT, WVC and the carrier-based technique. All warm obturation techniques demonstrated significantly greater sealer penetration effectiveness compared to the single-cone method. Additionally, the authors observed that dentinal tubule penetration was most pronounced in the coronal third of the root canal and least evident in the apical third.

Mobayed et al. (2024) [13] assessed the maximum depth of dentinal tubule penetration (DTPB) by the EndoSequence BC Sealer HiFlow (Brasseler, Savannah, GA, USA) sealer in three segments of the root canal: coronal, middle, and apical. Three obturation techniques were employed: the single cone (SC), cold lateral condensation (CLC), and WVC. In the coronal third, the WVC group exhibited the highest mean DTPB values, which were significantly greater than those observed in the CLC and SC groups. Similarly, in the middle third, the WVC group demonstrated superior sealer penetration compared to the CLC and SC groups. In contrast, in the apical third, the greatest penetration was noted in the CLC group, exceeding the values recorded in both the WVC and SC groups. Overall, the warm obturation techniques WVC and CLC showed statistically significantly better dentinal tubule penetration of the BCH sealer at all levels of the root canal compared to the single-cone technique.

The study by Reynolds et al. (2020) [14] compared the dentinal tubule penetration ability of three sealers, one resin-based 2Seal easymiX (Roydent, Johnson City, TN, USA) and two bioceramic sealers—EndoSequence BC Sealer (Brasseler, Savannah, GA, USA) and EndoSequence BC Sealer HiFlow (Brasseler, Savannah, GA, USA) using two obturation techniques: SC and WVC. Both the maximum penetration depth (in mm) and the percentage of the canal wall surface covered by the sealer were evaluated at 3 mm and 6 mm from the apex. At the 3 mm level, the BCH sealer exhibited superior tubule penetration compared to both the BC sealer and the resin-based sealer, regardless of the obturation technique used. However, at the 6 mm level, the BC sealer showed the greatest ability to fill the lateral canals. In all cases, the WVC technique yielded better outcomes than the SC technique.

The study by Merfea et al. (2024) [15] aimed to evaluate the push-out bond strength (POBS) of three different endodontic sealers: the resin-based AH Plus (Dentsply Sirona, Charlotte, NC, USA), the bioceramic TotallFill HiFlow (FKG Dentaire, Le Crêt-du-Locle, Switzerland), and the bioceramic AH Bio (Dentsply Sirona, Charlotte, NC, USA), using two obturation techniques, SC technique and WVC. The analysis of POBS revealed significant differences among the experimental groups. AH Plus demonstrated the highest adhesion values, regardless of the obturation technique employed. TotallFill HiFlow (FKG Dentaire, Le Crêt-du-Locle, Switzerland) exhibited significantly lower bond strength, while AH Bio (Dentsply Sirona, Charlotte, NC, USA) showed the weakest performance. The obturation technique did not significantly affect the POBS values within each sealer group, suggesting that the outcomes were primarily influenced by the intrinsic properties of the materials rather than the method of application. Nevertheless, AH Plus consistently demonstrated significantly higher POBS values across all sections of the root canal (apical, middle, and coronal), outperforming both AH Plus (Dentsply Sirona, Charlotte, NC, USA) and TotallFill HiFlow (FKG Dentaire, Le Crêt-du-Locle, Switzerland). Among the bioceramic sealers, TotallFill HiFlow (FKG Dentaire, Le Crêt-du-Locle, Switzerland) showed superior resistance to dislodgement from dentin compared to AH Bio, particularly in the middle and coronal thirds of the canal. Vemuri et al. (2024) [16] evaluated the influence of sealer type and obturation technique on the push-out bond strength (POBS) to root canal dentin. Two sealers—AH Plus Bioceramic (Dentsply Sirona, Charlotte, NC, USA) and EndoCeramic (Endo Direct) were tested in combination with three obturation techniques: cold lateral compaction, carrier-based technique, and thermoplasticized technique. The highest bond strength was observed with the combination of EndoCeramic (Endo Direct) and cold lateral compaction. However, the warm obturation techniques also demonstrated favorable adhesive properties. Within the carrier-based technique, the use of EndoCeramic (Endo Direct) resulted in significantly higher POBS values compared to the combination with AH Plus Bioceramic (Dentsply Sirona, Charlotte, NC, USA), confirming the efficacy of this approach in achieving strong adhesion to dentinal surfaces. Although the differences between the sealers in the thermoplasticized technique did not reach statistical significance, EndoCeramic (Endo Direct) still yielded superior results compared to AH Plus Bioceramic.

In the study by Yadav et al. (2020) [17], three obturation techniques, CLC, CBT and SC, were compared in combination with EndoSequence BC sealer (Brasseler, Savannah, GA, USA), focusing on material adaptation to canal walls and the presence of voids. At 2 mm from the apex, no significant differences were observed among the techniques regarding the proportions of gutta-percha, sealer, and voids. At 5 mm and 8 mm levels, the CBT demonstrated a significantly higher proportion of gutta-percha and a lower proportion of sealer compared to both CLC and SC techniques. The highest percentage of voids was recorded in the cold lateral compaction group. Regardless of the technique used, coronal sections exhibited a greater proportion of voids than the middle and apical sections.

Tek et al. (2020) [18] conducted a study where the highest percentage of external voids was observed in the group where bulk-fill TotalFill BC (FKG Dentaire, Le Crêt-du-Locle, Switzerland) was used, while the lowest was recorded with Biodentine (Septodont, Saint-Maur-des-Fossés, France). The mean values of external voids decreased in the following order: TotalFill BC (bulk-fill) > gutta-percha + TotalFill BC > MTA (Angulus) > Biodentine. A similar trend was observed for internal voids, with the greatest proportion also found in the bulk-fill Total Fill BC group. The lowest number of internal voids was noted in the group using gutta-percha combined with Total Fill BC, which showed significantly better outcomes than the other groups, with the exception of Biodentine. The descending order of mean internal void percentages was as follows: Total Fill BC (bulk-fill) > MTA > Biodentine > gutta-percha + Total Fill BC.

In the study by Manjila et al. (2022) [19], Meta Cera (Meta Biomed, Cheongju, Republic of Korea) demonstrated the lowest level of apical microleakage among all materials analyzed, indicating its superior ability to achieve a hermetic seal of the root canal system. AH Plus (Dentsply Sirona, Charlotte, NC, USA) and Sealapex (Kerr Sybron Endo, Orange, CA, USA) sealers showed slightly inferior performance but still significantly outperformed the conventional zinc oxide-eugenol-based material, which exhibited the highest degree of leakage. Regardless of the sealer used, the thermoplasticized condensation technique significantly outperformed cold lateral compaction in reducing apical microleakage. These differences were statistically significant across all material groups, underscoring the superiority of warm obturation techniques in achieving effective endodontic sealing.

### 3.2. Obturation of Apical Delta

The study conducted by Chan et al. (2024) [20] assessed the extent of sealer penetration into artificially prepared lateral canals at three levels of the root: coronal, middle, and apical. The analysis revealed no statistically significant differences between the types of sealers used. However, significant differences were observed between the obturation techniques, particularly in the coronal third. The CW technique enabled statistically deeper sealer penetration compared to the SC technique, especially when the TotalFill HiFlow BC (FKG Dentaire, Le Crêt-du-Locle, Switzerland) system was employed. In the middle and apical thirds, no significant differences between the techniques were noted. Regardless of the material or technique used, the highest level of sealer penetration was generally achieved in the apical third, while the lowest was observed in the coronal third.

The study conducted by Juha et al. (2024) [21] revealed significant differences in the effectiveness of lateral canal obturation among the evaluated techniques using a BCHiF Sealer (Brasseler, Savannah, GA, USA). The highest percentage of lateral canal filling was observed in the group where the CWT was applied, with statistically significant differences noted at all assessed levels (3 mm, 5 mm, and 7 mm from the apex). The CWT proved more effective than the other methods both in the apical region and at more coronal levels. In comparison, the cold lateral compaction technique showed lower filling rates, although still significantly higher than the single-cone technique, which consistently yielded the lowest percentage of lateral canal filling across all sections analyzed.

In the analysis conducted by Ko et al. (2020) [22] using micro-computed tomography (µCT), no significant differences were found among the tested obturation techniques in terms of the proportion of cross-sections containing voids or the volume of main canal filling. Voids were present in all groups, but their occurrence did not differ statistically. However, the results of stereomicroscopic analysis differed, with the SC technique showing a significantly higher mean number and severity of voids compared to the other techniques. No significant differences were observed between the single cone with ultrasonic activation (SCU) and WVT groups regarding the number and evaluation of voids.

### 3.3. Fracture Resistance

Two in vitro studies evaluated the fracture resistance of teeth obturated using premixed bioceramic sealers in combination with different obturation techniques.

Lichaa et al. (2022) [23] assessed 22 extracted human mandibular incisors. Root canals were prepared using the E3 Azure rotary system (Endostar) to size 25/0.06, with 1 mL of 5.25% NaOCl irrigation after each file and final irrigation performed using 3 mL of 5.25% NaOCl and 3 mL of 17% EDTA. The teeth were obturated with Bio-C Sealer^®^ (Angelus, Londrina, Paraná, Brazil) either with the SC or WVCT. Periodontal ligament simulation was achieved using a wax layer before embedding the roots in acrylic resin. Fracture resistance was tested using a universal testing machine with a 5 mm spherical fixture to apply compressive load until fracture. The authors found no statistically significant difference in fracture resistance between SC and WVCT, indicating that heat application in WVCT did not reduce fracture resistance in mandibular incisors.

Al-Hiyasat et al. (2023) [24] reached different conclusions. They investigated 80 extracted mature mandibular premolars prepared with the Mtwo rotary system (VDW) to size 40/0.04, with irrigation using 2 mL 5.25% NaOCl at each file change and final irrigation using 1 mL of 17% EDTA, and 5 mL of saline. Teeth were obturated with either TotalFill BC sealer (FKG Dentaire, Le Crêt-du-Locle, Switzerland) and coated bioceramic cones or AH Plus sealer (Dentsply Sirona, Charlotte, NC, USA) and gutta-percha, using SC, CLC or WVC techniques. Periodontal ligament simulation was achieved using silicone in acrylic blocks. Fracture resistance testing revealed that obturation significantly increased fracture resistance compared with prepared but unfilled roots. TotalFill BC sealer yielded significantly higher fracture resistance than AH Plus across all techniques. Among techniques, SC showed the highest fracture resistance, followed by CLC, with WVC yielding the lowest values. WVC performed with gutta-percha alone (without sealer) significantly reduced fracture resistance. Additionally, TotalFill BC significantly increased the calcium-to-phosphorus ratio in root dentin, as confirmed by EDX analysis (Table 5).

### 3.4. RCT Retreatability

Five ex vivo studies evaluated the retrievability of obturation materials following root canal filling with premixed bioceramic sealers in combination with warm condensation techniques.

Hassan et al. (2022) [25] examined 75 single-rooted premolars obturated using TotalFill HiFlow (FKG Dentaire, Le Crêt-du-Locle, Switzerland) and WVC, comparing the efficacy of XP Finisher, XP Finisher R, and passive ultrasonic irrigation (PUI) in removing the obturation material. The authors found that none of the protocols completely removed filling remnants, but all supplementary techniques improved cleanliness compared with syringe irrigation. XPF and XPR achieved significantly higher cleaning efficiency than PUI in all root segments, with the apical third consistently showing the lowest cleanliness. No significant difference was observed between XPF and XPR performance.

Elzanaty et al. (2024) [26] assessed the retreatability of NeoSEALER Flo (Avalon Biomed Inc., Houston, TX, USA) in canals obturated using either the WVC or SC technique, evaluating the performance of the ProTaper and EdgeFile XR systems in 32 maxillary premolars. They reported that no retreatment protocol completely removed gutta-percha and sealer from any canal third. In SC-obturated canals, a higher proportion of calcium silicate-based sealer resulted in more residual debris than in WVC canals. EdgeFile XR outperformed ProTaper in the middle third of WVC fillings, while no difference was found between systems for SC fillings. Across all groups, the apical third showed the most debris. The authors concluded that the SC technique with calcium silicate sealers may complicate nonsurgical retreatment and negatively affect outcomes.

Valerio et al. (2024) [27] investigated 36 mandibular incisors, comparing the efficiency of material removal from oval canals filled with Bio-C Sealer (Angelus, Londrina, Paraná, Brazil) using the SC technique, a modified McSpadden technique, or CWT by using ultrasonic tips and Reciproc files. They found no significant differences between obturation techniques in terms of the amount of remaining filling material, dentin wear, or root canal transportation. However, thermoplastic techniques required longer retreatment times than the SC approach. In all groups, complete removal of filling material was not achieved.

Eid et al. (2021) [28] analyzed 40 mandibular incisors to assess the effectiveness of XP-endo Finisher R and manual H-file instrumentation in removing TotalFill BC (FKG Dentaire, Le Crêt-du-Locle, Switzerland) from oval canals, utilizing micro-CT imaging. Rotary instruments alone achieved a median total filling reduction of about 70%, but complete removal was not possible. Both supplementary methods improved cleanliness, with XP-endo Finisher R removing significantly more residual material than H-files.

Zhang et al. (2021) [29], in a study on 40 premolars with oval canals, compared the retreatability of HiFlow (FKG Dentaire, Le Crêt-du-Locle, Switzerland)-filled canals (using SC or WVC techniques) after 2 weeks and 6 months, using XP-endo Finisher R as the supplementary removal method. They found that retreatment efficiency was more influenced by storage time than obturation technique, with prolonged storage reducing removal efficiency. XP-endo Finisher R was effective in removing remaining material from oval canals.

### 3.5. Post-Obturation Healing

Four clinical studies evaluated periapical tissue healing following endodontic treatment using bioceramic sealers in combination with warm obturation techniques.

Spinelli et al. (2023) [30] conducted a prospective pilot study involving 38 teeth with various forms of endodontic treatment using AH Plus Bioceramic (Dentsply Sirona, Charlotte, NC, USA) in combination with the carrier-based Thermafil technique. Healing, tooth survival, and the occurrence of postoperative pain were assessed after 12 months. The study reported a healing rate of 81.6% and a tooth survival rate of 100%, with most cases showing either complete resolution or improvement of periapical lesions. The authors concluded that flowable premixed sealers combined with a warm carrier-based technique are clinically effective, but further research is needed to clarify the biological role of sealer extrusion in periapical bone regeneration.

Algar et al. (2025) [31], in a randomized clinical trial involving 60 non-vital teeth (premolars, molars, incisors, and canines), compared AH Plus (Dentsply Sirona, Charlotte, NC, USA) and NeoSealer Flo (Avalon Biomed Inc., Houston, TX, USA) used with warm gutta-percha via the WVC technique, evaluating healing and symptoms after 6 months. NeoSealer Flo was associated with lower postoperative pain at 24 h and 7 days compared to AH Plus, while both sealers achieved similar periapical lesions healing rates. Material extrusion did not significantly influence pain or healing. The authors concluded that NeoSealer Flo offers advantages in early pain reduction without compromising healing efficacy, supporting its use as a bioactive alternative to conventional epoxy resin sealers.

Pontoriero et al. (2023) [32] evaluated 210 teeth, both primary treatments and retreatments, in the maxilla and mandible, using four different bioceramic sealers—CeraSeal (Meta Biomed, Cheongju, Republic of Korea), BioRoot (Septodont, Saint-Maur-des-Fossés, France), AH Plus Bio (Dentsply Sirona, Charlotte, NC, USA), and Bio-C SEALER ION (Angelus, Londrina, Paraná, Brazil)—in combination with either WVC or carrier-based obturation techniques. The overall success rate (healed and healing) was 99%, with all primary treatments achieving complete healing. Retreatment cases showed lower rates of complete healing (55.2%), but most were classified as healing (43.2%). The size of the initial periapical lesion influenced healing rates, with smaller lesions (<5 mm) healing more frequently than larger ones. Sealer extrusion did not affect overall success but was more often associated with cases still in the healing phase. The authors concluded that warm gutta-percha techniques with bioceramic sealers provide excellent clinical outcomes, and the presence of a periapical lesion does not compromise prognosis.

Zamparini et al. (2023) [33], in a two-year follow-up study of 94 root canal treatments- 47 performed with CeraSeal (Meta Biomed, Cheongju, Republic of Korea) and 47 with AH Plus (Dentsply Sirona, Charlotte, NC, USA) using the Thermafil technique. They evaluated periapical lesions healing, sealer extrusion, and tooth survival. Both sealers achieved similar healing and survival rates (~90%), with no new periapical lesions or re-exacerbations observed. Sealer extrusion did not negatively affect healing outcomes. The authors concluded that premixed CaSi-based sealers combined with warm carrier-based obturation are clinically effective and may offer biological advantages over epoxy-resin-based sealers.

### 3.6. Post-Obturation Complications

Three clinical studies provided data on complications associated with the use of premixed bioceramic sealers in combination with warm obturation techniques.

Bugea et al. (2022) [34] conducted a prospective study on 40 single-rooted teeth diagnosed with irreversible pulpitis, comparing four obturation techniques using different sealers, including EndoSequence BC Sealer (Brasseler, Savannah, GA, USA) combined with gutta-percha injection. Postoperative pain and treatment success rates were evaluated after one year. The authors found that the use of a bioceramic sealer was associated with significantly lower postoperative pain, as measured by reduced analgesic intake and absence of pain on percussion after one week, compared to zinc oxide eugenol sealers. Controlled thermoplastic injection minimized sealer extrusion and reduced the risk of periapical irritation.

Spinelli et al. (2023) [30] analyzed 40 root canals treated using the Thermafil technique with AH Plus Bioceramic (Dentsply Sirona, Charlotte, NC, USA), assessing postoperative pain and periapical lesions healing over a 12-month follow-up period. Sealer extrusion was radiographically detected in 47% of cases, but it did not significantly influence the healing rate at 12 months. Mild postoperative pain persisted in some patients during the first month and was associated with slower periapical lesions healing, although no direct link with extrusion was confirmed. The bioactive properties of the sealer may have mitigated potential adverse effects of extrusion.

Algar et al. (2025) [31], in a randomized clinical trial involving 60 non-vital teeth (incisors, canines, premolars, and molars), evaluated the frequency of sealer extrusion and the incidence of postoperative symptoms depending on the use of either AH Plus (Dentsply Sirona, Charlotte, NC, USA) or NeoSEALER Flo (Avalon Biomed Inc., Houston, TX, USA) in combination with the WVC technique and backfill. NeoSEALER Flo was associated with significantly lower postoperative pain within the first 24 h and at seven days compared to AH Plus, while both sealers showed similar periapical lesions healing rates after six months. Greater pain intensity was linked to sealer extrusion in both groups, but no significant difference in extrusion rates between sealers was observed.

## 4. Discussion

In recent years, there has been dynamic development in the field of endodontic obturation materials, particularly marked by growing interest in one-component premixed bioceramic sealers. These materials are available in a ready-to-use form, eliminating the need for mixing by the clinician, thereby reducing the risk of proportioning errors and increasing procedural consistency [35]. Their popularity is increasing not only in the traditional single-cone technique but also in combination with various warm obturation methods such as the CWT and thermomechanical compaction [36,37].

The main advantages of premixed bioceramic sealers include simplicity and speed of application, excellent biocompatibility, hydrophilicity, and the ability to set in a moist environment [38,39,40]. Additionally, they exhibit bioactive properties, promoting regenerative processes in the periapical tissues [41]. However, one of their major limitations is the relatively high cost, which may hinder routine clinical use, particularly in multi-rooted teeth where greater material volume is required [42,43].

A critical parameter determining the long-term success of root canal therapy is the sealability of the root canal filling [44,45]. In this context, premixed bioceramic sealers demonstrate favorable rheological properties, such as appropriate viscosity and the ability to penetrate narrow anatomical spaces [10,11,12,13,14,17,18]. This results in fewer voids, which could otherwise act as bacterial niches and contribute to reinfection [18,19].

Another important characteristic of these materials is their capacity to induce mineralization at the sealer–dentin interface. The adhesion of bioceramic sealers is primarily attributed to their ability to chemically interact with calcium ions in dentin and to promote hydroxyapatite deposition, which can effectively seal interfacial voids and microgaps. Upon contact with a moist canal environment, premixed bioceramic sealers facilitate the growth of hydroxyapatite crystals, leading to the formation of a biologically integrated interfacial layer between the material and the tooth structure [11]. This biomineralization process is considered to contribute to improved sealing and potential long-term dimensional stability of the root canal filling [46,47,48].

However, although the adhesive potential and mineralization ability of premixed bioceramic sealers are well documented, evidence regarding their microleakage behavior remains limited. Microleakage reflects the true sealing integrity of the root canal filling and depends not only on adhesion strength but also on the material’s dimensional stability and interfacial adaptation. Importantly, bond strength values alone cannot be directly extrapolated to predict microleakage, as leakage is governed by the continuity and integrity of the chemically and micromechanically integrated interfacial layer rather than by isolated adhesion strength measurements. Recent in vitro studies comparing TotalFill BC, Bio-C, and AH Plus sealers have reported heterogeneous results—some demonstrated comparable or reduced apical leakage for bioceramic sealers used with warm techniques, while others found increased leakage attributed to material shrinkage or incomplete adaptation after heating [47,49,50,51,52]. These discrepancies highlight the need for standardized microleakage evaluation protocols to confirm the long-term sealing performance of these materials.

The use of bioceramic-coated gutta-percha cones in the single-cone technique enables the formation of a so-called monoblock—a unified structure bonding the cone, sealer, and dentin. This configuration enhances mechanical sealing and limits microgaps [12,16]. However, there is a lack of data regarding the effectiveness of such cones in warm obturation techniques, where elevated temperatures may affect the material’s properties. Further research is necessary to assess these materials’ behavior under thermal conditions and their potential impact on sealing and bioactivity.

The incorporation of warm obturation techniques such as continuous wave condensation may further improve sealability [53]. Studies have shown that thermoplastic techniques enhance gutta-percha adaptation to canal walls and improve material conformance to anatomical irregularities compared to SC methods [13,14,15,16,17,54]. Therefore, combining bioceramic sealers with warm gutta-percha techniques may potentially synergize the benefits of both—bioceramic bioactivity and mineralization with the mechanical adaptation of gutta-percha. This represents a promising direction for modern endodontics, albeit one requiring further clinical validation.

Filling the apical delta and lateral canals remains a challenge due to their complex, irregular anatomy and often microscopic dimensions. Premixed bioceramic sealers, with their high flow and infiltration capacity, are particularly suited for penetrating even the smallest structures within the canal system, including dentinal tubules and lateral extensions [10,11]. This capability is further enhanced by warm gutta-percha techniques, which significantly improve material adaptation to canal walls and facilitate sealer flow into lateral canals and apical ramifications [14,20].

Mechanical instrumentation of such structures is generally impossible due to their narrow, irregular, and variable nature [55,56]. Hence, the cleaning of lateral canals relies primarily on the chemomechanical action of irrigating solutions, whose effectiveness can be enhanced through laser, sonic, or ultrasonic activation [57,58,59,60,61]. While activated irrigation effectively removes pulp remnants from difficult-to-access areas, it may also hinder complete canal drying—especially in lateral zones with limited accessibility and high humidity. In this context, the use of premixed bioceramic sealers—which not only tolerate moisture but require it for setting—becomes particularly justified [62]. In the presence of tissue fluids, these materials initiate hydroxyapatite formation, further sealing areas beyond mechanical reach and supporting tissue healing [11]. The ability to bioactively seal lateral canals and the apical delta makes bioceramics an attractive option in anatomically complex cases where complete obturation is essential for treatment success.

One of the most frequent long-term causes of endodontic failure leading to tooth extraction is root fracture [63]. This can result from both structural weakening during instrumentation and mechanical or thermal stress introduced during obturation [64]. Contemporary endodontics increasingly emphasizes the preservation of structural integrity, particularly the pericervical dentin (PCD), as critical for tooth longevity [65,66].

Thus, the obturation method—along with the coronal restoration technique—plays a significant role. Thermal obturation techniques such as CWT and WVC improve gutta-percha adaptation but can induce transient intradentinal temperature elevations. Sudden thermal fluctuations may cause microscopic stress and deformation within the dentin, potentially leading to crack formation and eventual fracture [67,68,69]. Additionally, mechanical forces during compaction, especially with vertical techniques can stress canal walls, particularly in teeth with thin roots or extensive preparation [70].

Premixed bioceramic sealers may help mitigate these effects. Due to their density and heat-absorbing properties, they act as thermal buffers, protecting dentin from abrupt temperature spikes [71]. Furthermore, these materials demonstrate good thermal stability under clinical conditions—temperatures generated during CWT rarely exceed 58 °C, a range tolerated by most bioceramics without compromising mechanical or adhesive properties [72].

Evidence on fracture resistance suggests that the impact of obturation technique is strongly influenced by the type of sealer used. Overall, bioceramic sealers appear to provide greater reinforcement of root dentin compared with epoxy resin-based sealers, particularly when combined with thermoplastic techniques. While warm vertical compaction may pose risks if performed without adequate buffering, its use alongside thermally stable bioceramic sealers does not seem to compromise structural integrity, even in anatomically delicate teeth [23]. Moreover, the enhanced mineralization and improved dentin crystallinity observed with bioceramic sealers indicate a potential protective effect against long-term weakening [24]. These findings highlight the need to carefully select both sealer type and obturation technique to preserve post-treatment mechanical stability, with particular attention to root anatomy, dentin thickness, and the desired long-term prognosis [73,74,75].

Even the most thoroughly performed endodontic treatments may fail due to reinfection, lateral canals, complex anatomy, or technical limitations [76]. In such cases, retreatment becomes necessary, and its effectiveness depends largely on the material initially used [77].

Premixed sealers like Bio-C Sealer or TotalFill BC exhibit strong dentin adhesion and deep tubular penetration, making removal difficult [78,79]. Retreatment studies consistently demonstrate that premixed bioceramic sealers are difficult to remove, regardless of technique. Thermoplastic obturation tends to complicate retreatment, requiring longer working times and leaving larger volumes of residual material, particularly in apical regions [25,26,27,28]. Although supplementary instruments such as XP-endo Finisher R improve removal efficiency, complete elimination of filling remnants remains unattainable [28,29]. Retreatment outcomes are further influenced by factors such as sealer age, with older materials showing greater removability, but even under favorable conditions substantial residues persist [29]. Collectively, these findings underscore a key clinical trade-off: while bioceramic sealers enhance sealing and long-term stability, they also present significant challenges for nonsurgical retreatment.

Despite advanced tools like XP-FR or ultrasonics, effective removal remains limited. The Self-Adjusting File (SAF) system may offer promise due to its mesh-like design, but its efficacy in removing thermally condensed bioceramics has yet to be evaluated [80,81,82].

Clinical evidence indicates that the combination of premixed bioceramic sealers with warm gutta-percha techniques yields consistently high rates of periapical lesions healing. Success rates commonly exceed 80–90%, independent of the specific sealer formulation or obturation method. Prognosis appears to be influenced more by baseline factors, such as lesion size or retreatment status, than by the material itself. Importantly, even in cases with sealer extrusion, healing outcomes were not compromised, reflecting the favorable biocompatibility of calcium silicate-based sealers. Overall, these findings suggest that bioceramic sealers provide reliable clinical performance when integrated with thermoplastic techniques, particularly in primary treatments [30,31,32,33].

These outcomes are likely due to bioceramic bioactivity. Calcium compounds induce alkalinity, promote hydroxyapatite formation, support dentin remineralization, and periapical tissue healing [83,84]. Bioceramic sealers stimulate osteoblasts and IL-10 expression, promoting anti-inflammatory and osteogenic responses [85,86,87,88]. Some cases show partial or complete apical foramen closure by mineralized tissue, potentially preventing reinfection [89].

Studies comparing various obturation techniques show that combining premixed sealers with warm gutta-percha provides at least comparable—and often superior—clinical outcomes due to the synergy of thermoplastic adaptation and bioceramic bioactivity [90]. This combination may be especially valuable in complex clinical cases, though long-term studies are needed.

Early endodontic complications associated with obturation materials include material extrusion and postoperative pain [15,91]. Premixed sealers, though praised for their flow and penetrability, are also more prone to unintentional extrusion due to their small particle size (e.g., ~2 µm in EndoSequence BC Sealer) and syringe-based delivery, especially with wide apices or excessive pressure [92,93]. Accurate working length and apical control are therefore essential [94,95].

Sealer extrusion appears relatively frequent with premixed bioceramics, yet current evidence suggests it has minimal clinical impact [30,31]. Reported extrusion rates vary around one third of cases, but healing outcomes remain unaffected, likely due to the high biocompatibility and partial resorption of calcium silicate-based materials [96,97]. Similarly, postoperative pain is a common finding after root canal treatment, but bioceramic sealers tend to be associated with reduced early discomfort compared to epoxy resin-based sealers [98,99]. While extrusion may transiently intensify pain in the immediate postoperative period, overall symptoms are typically mild, self-limiting, and not detrimental to long-term healing [30,31,34]. Long-term clinical and radiographic follow-ups have demonstrated that extruded material does not adversely affect healing outcomes and often remains stable or undergoes partial resorption over time [98,100].

Additionally, endodontic pain usually responds well to NSAIDs, making it a manageable concern rather than a clinical limitation [101,102,103].

The interaction of premixed calcium silicate-based sealers with tissue fluids also requires clarification. Studies have shown that these materials exhibit partial solubility and ionic exchange when immersed in aqueous or simulated body fluid environments, leading to ion release, surface dissolution, and subsequent calcium phosphate precipitation [104,105,106,107]. This controlled dissolution is considered an integral part of their bioactive mechanism, facilitating hydroxyapatite nucleation and chemical bonding with dentin. However, excessive solubility could compromise dimensional stability and long-term sealing, particularly in overextended fillings or perforation sites. The bioactivity and sealing ability of premixed bioceramic sealers have therefore prompted consideration of their use in managing root canal or root perforations; nevertheless, their partial resorbability in tissue fluids raises concerns about long-term stability in these indications. Consequently, MTA-based materials remain the gold standard for perforation repair due to their superior durability and clinical predictability, and further clinical research is needed to define safe indications for premixed bioceramic sealers in defect management [106,108].

## 5. Conclusions

The use of premixed bioceramic sealers in combination with warm gutta-percha techniques represents a promising strategy in root canal obturation, merging the advantages of bioactive materials with enhanced mechanical adaptation. Based predominantly on in vitro data and a limited number of short-term clinical studies, this approach appears to offer excellent sealability, mineralization potential, and favorable biological properties that may support periapical healing. However, clinicians must be mindful of potential retreatment challenges and extrusion risks, and current evidence should be interpreted with caution. Careful case selection, precise instrumentation, and a thorough understanding of sealer properties are essential, while long-term, well-designed clinical studies are still needed to confirm the durability and prognostic impact of this modern endodontic approach.

## 6. Limitations

This review has several limitations that must be acknowledged. Most of the included studies were conducted under in vitro or ex vivo conditions, which—despite their methodological value—do not fully reflect clinical reality, particularly regarding tissue healing and immune response. Moreover, the reviewed literature was characterized by significant heterogeneity in terms of materials, obturation techniques, evaluation methods, and outcome measures, making direct comparisons difficult and preventing data pooling or meta-analysis. Although several clinical studies were included, the majority had short follow-up periods, limiting the assessment of long-term outcomes such as durability, healing, or complications. Consequently, robust evidence on the long-term prognostic impact of premixed bioceramic sealers used with warm obturation techniques remains insufficient. Another important gap concerns retreatability: while some in vitro studies have assessed the removal of premixed bioceramic sealers from canals filled with warm gutta-percha, high-quality clinical data on retreatment feasibility, procedural safety, and long-term success rates are still lacking. Additionally, only articles published in English and indexed in selected databases were included, which may introduce language and publication bias.

Future studies should therefore focus on standardized protocols, extended follow-up periods, and clinically relevant retreatment outcomes to more reliably define the role of premixed bioceramic sealers combined with warm obturation techniques in routine endodontic practice.

## Figures and Tables

**Table 1:** Inclusion and exclusion criteria for studies on sealing, obturation, fracture resistance and RCT retreatability shown using the PICO framework.

Parameter	Inclusion Criteria	Exclusion Criteria
Population (P)	in vitro studies using removed human teeth	3D-printed teeth models
Intervention(I)	warm obturation techniques	cold obturation techniques only
Comparison (C)	comparison of BC and other endodontic sealants comparison of warm obturation techniques to SC and CLC	–
Outcomes (O)	evaluation of root canal treatment obturation quality, mechanical properties, accuracy of retreatment	–
Study Design	clinical studies, cohort and cross-sectional studies published after 2020	systematic reviews, case reports, conference reports, editorials, works not published in English, non-human studies

**Table 2:** Inclusion and exclusion on post-obturation healing and complications criteria shown using the PICO framework.

Parameter	Inclusion Criteria	Exclusion Criteria
Population (P)	in vivo studies on human populations	in vitro studies
Intervention(I)	warm obturation techniques	cold obturation techniques only
Comparison (C)	comparison of BC and other endodontic sealants comparison of warm obturation techniques to SC and CLC	–
Outcomes (O)	healing of lesions and postoperative complications	–
Study Design	clinical studies, cohort and cross-sectional studies published after 2020	systematic reviews, case reports, conference reports, editorials, works not published in English, non-human studies

**Table 3:** General characteristics of included studies: Physicochemical Properties and Sealing Effectiveness.

Author and Year	Journal	Study Type	Obturation Techniques	Materials	Teeth Examined	Objective	Results
Abu-Zeid et al. (2024) [10]	Journal of Functional Materials	in vitro	(1) vertical condensation (VC) (2) warm vertical compaction (WVC)	EndoSequence BC sealer (Brasseler, Savannah, GA, USA), EndoSequence BC Sealer HiFlow (Brasseler, Savannah, GA, USA), BC points (Brasseler, Savannah, GA, USA), AH Plus (Dentsply Sirona, Charlotte, NC, USA), gutta-percha (ND)	54 (extracted mandibular premolars)	To evaluate the adaptability and intratubular penetration depth of bioceramic systems using different obturation techniques.	EndoSequence BC Sealer HiFlow (Brasseler) sealer use resulted in the greatest dentinal tubule penetration.
Alsofi (2024) [11]	Bioactivity of Endodontic Sealers	in vitro	(1) single-cone (SC) technique (2) warm vertical compaction (WVC)	EndoSequence BC sealer (Brasseler, Savannah, GA, USA), EndoSequence BC Sealer HiFlow (Brasseler, Savannah, GA, USA), AH Plus (Dentsply Sirona, Charlotte, NC, USA), gutta-percha (ND)	0 (extracted mandibular premolars)	To assess the bioactivity and intratubular penetration depth of three different endodontic sealers applied with a warm compaction technique and single cone technique	EndoSequence BC Sealer HiFlow (Brasseler) sealer use resulted in the greatest dentinal tubule penetration. Furthermore, incubating the BC Sealer HiFlow in phosphate-buffered saline (PBS) led to the formation of hydroxyapatite crystal
Casino-Alegre et al. (2023) [12]	Journal of Clinical and Experimental Dentistry	in vitro	(1) single-cone (SC) technique (2) continuous wave technique (CWT) (3) vertical condensation (VC) (4) carrier-based technique	EndoSequence BC Sealer HiFlow (Brasseler, Savannah, GA, USA), GuttaCore (Dentsply Sirona, Charlotte, NC, USA), gutta-percha (Dentsply Sirona, Charlotte, NC, USA), gutta-percha (TotalFill BC, FKG Dentaire, Le Crêt-du-Locle, Switzerland)	180 (extracted incisors, canines and premolars)	To assess the intratubular penetration percentage of the calcium silicate-based sealer along the canal perimeter using three different warm obturation techniques	Warm obturation techniques exhibited higher intratubular penetration percentage compared to the SC.
Manjila et al. (2022) [19]	Journal of Conservative Dentistry	in vitro	(1) cold lateral compaction (2) undisclosed thermoplasticized technique	Tubli-Seal (Sybron Endo), Sealapex (Kerr Sybron Endo, Orange, CA, USA), AH Plus (Dentsply Sirona, Charlotte, NC, USA), Meta Cera (Meta Biomed, Cheongju, Republic of Korea)	96 (extracted mandibular premolars)	To assess apical microleakage in root canals containing fractured rotary instruments, obturated using cold lateral condensation and thermoplasticized injectable gutta-percha techniques with various sealers	The thermoplasticized condensation method consistently outperformed CLC in minimizing apical microleakage, irrespective of the sealer type.
Merfea et al. (2024) [15]	Dentistry Journal	in vitro	(1) single-cone (SC) technique (2) continuous wave technique (CWT)	AH Plus Bioceramic Sealer (Dentsply Sirona, Charlotte, NC, USA), AH Plus (Dentsply Sirona, Charlotte, NC, USA), TotalFill HiFlow BC (FKG Dentaire, Le Crêt-du-Locle, Switzerland), gutta-percha (Dentsply Sirona, Charlotte, NC, USA)	36 (extracted mono-radicular teeth)	To assess the push-out bond strength (POBS) of calcium silicate-based sealers in comparison with an epoxy resin-based sealer using various obturation techniques	AH Plus exhibited consistently greater POBS values in the apical, middle, and coronal regions of the root canal, surpassing both AH Plus Bioceramic (Dentsply Sirona) and TotalFill HiFlow (FKG Dentaire).
Mobayed et al. (2024) [13]	Cureus	in vitro	(1) single-cone (SC) technique (2) cold lateral compaction (CLC) (3) warm vertical compaction (WVC)	EndoSequence BC Sealer HiFlow (Brasseler, Savannah, GA, USA)	45 (extracted mandibular premolars)	To evaluate the extent of dentinal tubule penetration achieved by a bioceramic sealer	Warm obturation techniques exhibited higher intratubular penetration percentage compared to the SC.
Reynolds et al. (2020) [14]	Australian Endodontic Journal	in vitro	(1) single-cone (SC) technique (2) warm vertical compaction (WVC)	2Seal easymiX (Roydent, Johnson City, TN, USA), EndoSequence BC Sealer HiFlow (Brasseler, Savannah, GA, USA), gutta-percha (ND)	50 (extracted incisors, canines and premolars)	To assess the depth and percentage of dentinal tubule infiltration achieved by two bioceramic sealers when applied using different obturation techniques	WVC exhibited higher intratubular penetration percentage compared to the SC.
Tek et al. (2020) [18]	Restorative Dentistry and Endodontics	in vitro	(1) warm vertical compaction (WVC)	White MTA (Angelus, Londrina, Paraná, Brazil), Biodentine (Septodont, Saint-Maur-des-Fossés, France), TotalFill BC (FKG Dentaire, Le Crêt-du-Locle, Switzerland)	40 (extracted maxillary central incisors)	To evaluate the obturation quality of various materials combined with warm gutta-percha (WGP) in teeth with simulated internal root resorption (IRR)	The fewest internal voids were observed in specimens obturated with gutta-percha in combination with TotalFill BC (FKG Dentaire, Le Crêt-du-Locle, Switzerland).
Vemuri et al. (2024) [16]	Journal of Conservative Dentistry and Endodontics	in vitro	(1) cold lateral compaction (CLC) (2) carrier-based technique (CBT) (3) thermoplasticized technique	AH Plus Bioceramic Sealer (Dentsply Sirona, Charlotte, NC, USA), EndoCeramic Bioceramic Sealer (Endo Direct)	42 (extracted single rooted, no specific data)	To investigate the impact of different obturation techniques on the push-out bond strength (POBS) of various endodontic sealers	Warm obturation techniques also demonstrated higher POBS. No differences between AH Plus Bioceramic Sealer (Dentsply Sirona) and EndoCeramic Bioceramic Sealer (Endo Direct)
Yadav et al. (2020) [17]	European Endodontic Journal	in vitro	(1) single-cone (SC) technique (2) cold lateral compaction (CLC) (3) carried-based technique (CBT)	EndoSequence BC sealer (Brasseler, Savannah, GA, USA), GuttaCore (Dentsply Sirona, Charlotte, NC, USA), gutta-percha (Dentsply Sirona, Charlotte, NC, USA), CPoint (EndoTechnologies)	90 (extracted mandibular incisors)	To compare the quality of root canal obturation achieved using various techniques	CBT achieved the highest quality of root canal filling. In contrast, SC was associated with internal defects, including tears and delamination, which may compromise its long-term performance.

**Table 4:** General characteristics of included studies: Obturation of Apical Delta.

Author and Year	Journal	Study Type	Obturation Techniques	Materials	Teeth Examined	Objective	Results
Chan et al. (2024) [20]	International Dental Journal	in vitro	(1) single-cone (SC) technique (2) wave compaction (CW)	AH Plus (Dentsply Sirona, Charlotte, NC, USA), iRoot SP (Innovative BioCeramix.), TotalFill HiFlow BC (FKG Dentaire, Le Crêt-du-Locle, Switzerland), gutta-percha (Dentsply Sirona, Charlotte, NC, USA)	90 (30 maxillary incisors, 60 mandibular premolars)	To compare the penetration efficacy of calcium silicate-based sealers and an epoxy resin-based sealer into lateral canals using either the single-cone or continuous wave compaction obturation techniques	No statistical differences between the materials and techniques used.
Juha et al. (2024) [21]	BDJ Open	in vitro	(1) single-cone (SC) technique (2) continuous wave technique (CWT) (3) cold lateral condensation (CLC)	BCHiF Sealer (Brasseler, Savannah, GA, USA), gutta-percha (DiaDent)	30 (mandibular premolars)	To assess the efficacy of simulated lateral canal obturation using various techniques, including continuous wave compaction, cold lateral condensation, and the single cone method	CWT was more effective than the alternative approaches at both apical and coronal levels. Although CLC resulted in reduced filling quality, it still outperformed SC.
Ko et al. (2020) [22]	Materials	in vitro	(1) single-cone (SC) technique (2) single cone with ultrasonic activation (SCU) (3) warm vertical compaction (WVC)	Endoseal TCS (MARUCH), gutta-percha (DiaDent)	105 (ND)	To assess the efficacy of various obturation techniques influence the filling quality of main and accessory canals when using a premixed calcium silicate sealer	There were no statistically significant differences between the SCU and WVT groups in terms of void number or distribution.

**Table 5:** General characteristics of included studies: Fracture Resistance.

Author and Year	Journal	Study Type	Obturation Techniques	Materials	Teeth Examined	Objective	Results
Al-Hiyasat et al. (2023) [24]	Clinical Oral Investigations	in vitro	(1) single-cone (SC) technique (2) cold lateral compaction (CLC) (3) warm vertical compaction (WVC)	TotalFill BC (FKG Dentaire, Le Crêt-du-Locle, Switzerland), AH Plus (Dentsply Sirona, Charlotte, NC, USA), gutta-percha (ND)	80 (extracted mandibular premolars)	To evaluate the effect of sealer type and obturation technique on root fracture resistance	SC showed the highest fracture resistance, followed by CLC, with WVC yielding the lowest values. WVC performed with gutta-percha alone significantly reduced fracture resistance
Lichaa et al. (2022) [23]	The Journal of Contemporary Dental Practice	in vitro	(1) single-cone (SC) technique (2) continuous wave technique (CWT)	Bio-C sealer (Angelus, Londrina, Paraná, Brazil), gutta-percha FM (Meta Biomed, Cheongju, Republic of Korea)	22 (extracted mandibular incisors)	To compare fracture resistance of single-cone vs. warm vertical compaction technique	No statistically significant difference in fracture resistance between SC and CWT.

**Table 6:** General characteristics of included studies: RCT Retreatability.

Author and Year	Journal	Study Type	Obturation Techniques	Materials	Teeth Examined	Objective	Results
Eid et al. (2021) [28]	Giornale Italiano di Endodonzia	ex vivo	(1) warm vertical compaction (WVC)	TotalFill BC (FKG Dentaire, Le Crêt-du-Locle, Switzerland), gutta-percha (Dentsply Sirona, Charlotte, NC, USA)	40 (mandibular incisors)	Evaluate efficacy of XP-endo Finisher-R and manual H-filing in removing bioceramic sealer from oval canals using micro-CT	Rotary instruments alone achieved a median total filling reduction; XP-endo Finisher R removed significantly more residual material than H-files.
Elzanaty et al. (2024) [26]	Journal of Conservative Dentistry and Endodontics	ex vivo	(1) single-cone (SC) technique (2) warm vertical compaction (WVC)	NeoSEALER Flo (Avalon Biomed Inc., Houston, TX, USA), gutta-percha (ND)	32 (maxillary premolars)	Compare retreatability of NeoSEALER Flo obturated with WVC vs. single-cone using ProTaper or EdgeFile XR	No complete removal: SC promote more residue than WVC; EdgeFile XR is better than ProTaper (in the middle third WVC); most debris in apical third.
Hassan et al. (2022) [25]	European Endodontic Journal	ex vivo	(1) warm vertical compaction (WVC)	TotalFill BC (FKG Dentaire, Le Crêt-du-Locle, Switzerland), gutta-percha EQ-V (Cerkamed)	75 (single-rooted premolars)	Compare cleaning efficiency of XP Finisher, XP Finisher R, and PUI after retreatment	No complete removal: XPF, XPR is better than PUI in all segments; apical third least clean.
Valerio et al. (2024) [27]	Clinical Oral Investigations	ex vivo	(1) single-cone (SC) technique (2) warm vertical compaction (WVC)	Bio-C (Angelus, Londrina, Paraná, Brazil), gutta-percha (Maillefer Instruments SA)	36 (mandibular incisors)	Evaluate effect of obturation technique on filling removal efficiency in oval canals	No differences between techniques; thermoplastic takes longer time; no complete removal.
Zhang et al. (2021) [29]	Clinical Oral Investigations	ex vivo	(1) single-cone (SC) technique (2) warm vertical compaction (WVC)	BC HiFlow (ND), gutta-percha Reciproc (VDW)	40 (premolars with oval canals)	Assess voids/gaps and retreatability after 2 weeks or 6 months using SCO or WVC with XP-endo Finisher R as supplementary step	Longer storage—lower efficiency; XPR is effective in oval canals.

**Table 7:** General characteristics of included studies: Post-obturation Healing.

Author and Year	Journal	Study Type	Obturation Techniques	Materials	Teeth Examined	Objective	Results
Algar et al. (2025) [31]	Bioengineering	Randomized clinical trial	(1) warm vertical compaction (WVC) (2) thermoplastic backfill	Neosealer Flo (Avalon Biomed Inc., Houston, TX, USA), AH Plus (Dentsply Sirona, Charlotte, NC, USA), gutta-percha Superendo Alpha (B&L)	60 necrotic teeth (premolars, molars, incisors, canines)	To compare the effects of AH Plus and Neosealer Flo on postoperative pain and healing of periapical lesions over a 6-month period.	Both sealers achieved similar periapical lesions healing rates after 6 months.
Pontoriero et al. (2023) [32]	Journal of Clinical Medicine	Prospective clinical study	(1) continuous wave (CWT) (2) carrier-based technique (CBT)	CeraSeal (Meta Biomed, Cheongju, Republic of Korea), BioRoot (Septodont, Saint-Maur-des-Fossés, France), AH Plus Bioceramic (Dentsply Sirona, Charlotte, NC, USA), gutta-percha (ND)	210 (98 primary RCT, 112 retreatment; maxillary and mandibular anterior and posterior teeth)	To evaluate the clinical outcomes of endodontically treated teeth obturated with various bioceramic sealers and warm gutta-percha techniques over a minimum of 18 months.	Overall success rate (healed + healing) 99%; complete healing in all primary RCTs, 55.2% in retreatments; smaller lesions (<5 mm) healed more frequently.
Spinelli et al. (2023) [30]	Applied Sciences	Prospective clinical (pilot)	(1) carrier-based technique (CBT)	AH Plus Bioceramic (Dentsply Sirona, Charlotte, NC, USA), gutta-percha Thermafill (Dentsply Sirona, Charlotte, NC, USA)	38 (maxillary and mandibular anterior and posterior teeth with endodontic pathology)	To evaluate the 12-month clinical outcomes of teeth obturated with AH Plus Bioceramic sealer and warm carrier-based technique, including healing, survival rate, and post-operative pain.	Healing rate after 12 months was 81.6%, with most lesions showing resolution or improvement.
Zamparini et al. (2023) [33]	Journal of Functional Biomaterials	Prospective cohort study	(1) carrier-based technique (CBT)	Ceraseal (premixed CaSi-based bioceramic sealer) AH Plus (Dentsply Sirona, Charlotte, NC, USA), gutta-percha Thermafill (Dentsply Sirona, Charlotte, NC, USA)	94 (primary RCT, retreatment; maxillary and mandibular anterior and posterior teeth)	To evaluate and compare the 2-year clinical outcomes of Ceraseal vs. AH Plus used with warm carrier-based technique, focusing on periapical lesions healing, sealer extrusion, and survival.	Healing rate after 2 years was ~90% for both sealers; no new lesions observed.

**Table 8:** General characteristics of included studies: Post-obturation Complications.

Author and Year	Journal	Study Type	Obturation Techniques	Materials	Teeth Examined	Objective	Results
Algar et al. (2025) [31]	Bioengineering	Randomized clinical trial	(1) warm vertical compaction (WVC) (2) thermoplastic backfill	Neosealer Flo (Avalon Biomed Inc., Houston, TX, USA), AH Plus (Dentsply Sirona, Charlotte, NC, USA), gutta-percha Superendo Alpha (B&L)	60 necrotic teeth (premolars, molars, incisors, canines)	To compare postoperative pain, extrusion, and periapical lesions healing after root canal obturation using AH Plus or Neosealer Flo sealers.	NeoSEALER Flo caused significantly lower pain at 24 h and 7 d vs. AH Plus. Greater pain intensity was associated with extrusion, but extrusion rates were similar between groups.
Bugea et al. (2022) [34]	Journal of Osseointegration	Prospective clinical study	(1) single cone (SC) (2) thermoplastic gutta-percha injection	EndoSequence BC (Brasseler, Savannah, GA, USA), gutta-percha (Brasseler, Savannah, GA, USA), gutta-percha (Dentsply Sirona, Charlotte, NC, USA), gutta-percha Thermafill (Dentsply Sirona, Charlotte, NC, USA)	40 (single-rooted teeth with irreversible pulpitis, ND)	To evaluate postoperative pain and 1-year success rate of four obturation techniques using different sealers in single-rooted teeth.	Bioceramic sealer group had lower postoperative pain (less analgesic use, no percussion pain after 1 week) vs. ZOE sealers. Controlled thermoplastic injection minimized extrusion and periapical irritation.
Spinelli et al. (2023) [30]	Applied Sciences	Prospective clinical pilot study	(1) carrier-based technique (CBT)	AH Plus Bioceramic (Dentsply Sirona, Charlotte, NC, USA), gutta-percha Thermafill (Dentsply Sirona, Charlotte, NC, USA)	38 (maxillary and mandibular anterior and posterior teeth with endodontic pathology)	To evaluate the 12-month periapical lesions healing, survival rate, and post-operative pain associated with a novel premixed CaSi-containing sealer used with warm carrier-based obturation.	Extrusion observed in 47% of cases, without significant effect on healing. Mild pain persisted in some patients during the first month, linked to slower healing but not directly to extrusion.

## Data Availability

Data is contained within the article.

## Supplementary Materials

The following supporting information can be downloaded at: https://www.mdpi.com/article/10.3390/gels11110932/s1: Reporting checklist for systematic review (with or without a meta-analysis).

## Abbreviations

The following abbreviations are used in this manuscript:

ND	No Data
PRISMA	Preferred Reporting Items for Systematic Reviews and Meta-Analyses
RCT	Root Canal Treatment
BC	Bioceramic Sealer
BCH	Bioceramic HiFlow Sealer
SC	Single Cone Technique
SCU	Single Cone with Ultrasound Activation
CLC	Cold Lateral Condensation
VC	Vertical Compaction
WVC	Warm Vertical Compaction
CWT	Continuous Wave Technique
CBT	Carrier-Based Technique
GP	Gutta-Percha
PBS	Phosphate-buffered Saline
DTPB	Dentinal Tubule Penetration of Bioceramic Sealer
PUI	Passive Ultrasonic Irrigation
PCD	Pericervical Dentin
SAF	Self-Adjusting File
